# Qualitative study of the acceptability and feasibility of acceptance and commitment therapy for adolescents with chronic fatigue syndrome

**DOI:** 10.1136/bmjpo-2021-001139

**Published:** 2021-10-01

**Authors:** Philippa Clery, Jennifer Starbuck, Amanda Laffan, Roxanne Morin Parslow, Catherine Linney, Jamie Leveret, Esther Crawley

**Affiliations:** 1 Centre for Academic Child Health, University of Bristol, Bristol, UK; 2 Paediatric CFS/ME Service, Royal United Hospital Bath NHS Trust, Bath, UK

**Keywords:** psychology, qualitative research, adolescent health

## Abstract

**Background:**

Paediatric chronic fatigue syndrome/myalgic encephalomyelitis (CFS/ME) is disabling and relatively common. Although evidenced-based treatments are available, at least 15% of children remain symptomatic after one year of treatment. Acceptance and commitment therapy (ACT) is an alternative therapy option; however, little is known about whether it is an acceptable treatment approach. Our aim was to find out if adolescents who remain symptomatic with CFS/ME after 12 months of treatment would find ACT acceptable, to inform a randomised controlled trial (RCT) of ACT.

**Methods:**

We recruited adolescents (diagnosed with CFS/ME; not recovered after one year of treatment; aged 11–17 years), their parent/carer and healthcare professionals (HCPs) from one specialist UK paediatric CFS/ME service. We conducted semi-structured interviews to explore barriers to recovery; views on current treatments; acceptability of ACT; and feasibility of an effectiveness RCT. Thematic analysis was used to identify patterns in data.

**Results:**

Twelve adolescents, eleven parents and seven HCPs were interviewed. All participants thought ACT was acceptable. Participants identified reasons why ACT might be efficacious: pragmatism, acceptance and compassion are valued in chronic illness; values-focussed treatment provides motivation and direction; psychological and physical needs are addressed; normalising difficulties is a useful life-skill. Some adolescents preferred ACT to cognitive behavioural therapy as it encouraged accepting (rather than challenging) thoughts. Most adolescents would consent to an RCT of ACT but a barrier to recruitment was reluctance to randomisation. All HCPs deemed ACT feasible to deliver. Some were concerned patients might confuse ‘acceptance’ with ‘giving up’ and called for clear explanations. All participants thought the timing of ACT should be individualised.

**Conclusions:**

All adolescents with CFS/ME, parents and HCPs thought ACT was acceptable, and most adolescents were willing to try ACT. An RCT needs to solve issues around randomisation and timing of the intervention.

**What is known about the subject?**

- Not all young people with chronic fatigue syndrome/myalgic encephalomyelitis (CFS/ME) recover.
- Acceptance and commitment therapy (ACT) is a possible alternative therapy for CFS/ME, which focuses on improving functioning and quality of life rather than symptom reduction.
- ACT is efficacious in paediatric chronic pain, and preliminary results show promising effects in adults with CFS/ME.

**What this study adds?**

- ACT is an acceptable therapy for young people with CFS/ME.
- Participants thought the ‘pragmatic’, ‘compassionate’ and ‘values-based’ focus of ACT would be helpful.
- Adolescents, parents and healthcare professionals support a randomised controlled trial of ACT.

## Introduction

Paediatric chronic fatigue syndrome/myalgic encephalomyelitis (CFS/ME) is relatively common (prevalence 0.55% across community, primary care and hospital populations)^1^ and can be severely disabling with persistent fatigue, chronic pain, postural instability and cognitive dysfunction.^2^ It negatively impacts on children’s emotional,^3–5^ educational^6^ and social functioning.^7^ Despite specialist treatments (cognitive behavioural therapy-for-fatigue (CBT-f), activity management (AM) and graded exercise therapy (GET)), at least 15% of children with CFS/ME remain symptomatic after one year of treatment.^8^ Alternative treatment approaches are needed.

Acceptance and commitment therapy (ACT) is an approach used in related conditions in children.^9 10^ A randomised controlled trial (RCT) in paediatric chronic pain suggests ACT is better than standard care at improving functional disability and health-related quality of life,^11^ and recent WHO guidelines recommend ACT for treating chronic pain in children.^12^ Studies of ACT in CFS/ME have focused on adults. One feasibility study in 40 adults with CFS/ME showed ACT resulted in sustained improvements in CFS/ME-related disability at 6 months.^13^

ACT offers a similar but different approach to CBT-f.^14^ Differences include: focussing on improving functioning and quality of life by aligning behaviour with chosen values, rather than reducing symptoms; stepping away from thoughts (cognitive defusion) rather than challenging them; and acting presently in the moment at whatever current functional capacity is possible (psychological flexibility).^15 16^

We aimed to determine if ACT is an acceptable treatment approach for adolescents who remain symptomatic after 12 months of treatment, and whether it would be an acceptable intervention for an effectiveness RCT of ACT.

## Methods

### Design

A qualitative study using a truth and reality-oriented approach^17^ to provide a real-world, multi-perspective view on ACT and a potential RCT of ACT versus treatment-as-usual.

### Recruitment

Participants were recruited from one UK specialist paediatric CFS/ME service. It was not deemed feasible to contact all eligible participants in the service, so sampling was opportunistic that is, individuals who had clinic appointments with a clinician or therapist in the CFS/ME service within the recruitment timeframe were approached. Inclusion criteria: adolescents (11–17 years) with CFS/ME,^2^ not recovered after one year of treatment (ie, ongoing care with the service); their parents; CFS/ME healthcare professionals (HCPs). Eligible participants were approached in clinic, given information leaflets and, if interested, provided consent to be contacted by the study lead (PC) who answered any questions and consented them into the study. Parents were eligible if their child was eligible and consented to participate. They were recruited alongside their child. HCPs were given information leaflets in a team meeting and via email, and if interested, consented into the study by contacting the study lead (PC).

### Data collection

Semi-structured interviews and one HCP focus group were undertaken (PC) February to September 2020 until data saturation was achieved.^18^ Participants were interviewed at home, the CFS/ME service or over Skype. From March 2020, all were over Skype due to the COVID-19 pandemic. Adolescents and parents were asked to be interviewed separately but were given the option to be together.

Topic guides (see online supplemental material) were developed with psychologists (JS, AL), a qualitative researcher (RMP), clinician (EC) and Young Person Advisory Group. Questions explored: treatment needs; acceptability of ACT; and trialling ACT. HCPs were asked additional questions on delivering ACT. Interviews were checked with an experienced qualitative researcher (RMP) to adapt topic guides, and monitor and improve interview technique. A standardised easy-to-understand explanation of ACT called ‘James’ Story’ (written by JS and AL) was provided written and orally to participants before and during the interview. It highlights the key elements of ACT and how it differs from CBT-f that participants may be more familiar with (see online supplemental material).

10.1136/bmjpo-2021-001139.supp1 Supplementary data

### Analysis

Interviews were recorded, transcribed, anonymised and imported into qualitative data-management software NVivo (PC). Notes were made during interviews. Transcripts were analysed using thematic analysis^19^ to identify patterns within the data. Transcripts were double-coded (CL, AL, JS, JL) and disagreements discussed. Deductive coding was used to create a coding framework around the pre-existing ‘sensitising concepts’^20^ of overarching themes ‘ACT acceptability’ and ‘trialling ACT’. Inductive coding was then used to derive codes from participants’ own words to provide more detail and generate subthemes. Data were checked between participants to explore the range of views.

## Results

### Participants

We interviewed 30 participants (online supplemental table 1): 12 adolescents (10 were female; age=12–17 years, median=15.5 years; in the service for 2–5 years) and 11 parents (10 were mothers; one was the parent of two adolescents). Of 14 adolescents approached, one declined to participate, one was ineligible. Three child-parent dyads were interviewed together, the remainder separately. We interviewed seven HCPs (clinicians, psychologists, physiotherapists and occupational therapists). Five took part in a focus group, two were interviewed individually. Interviews lasted 30–110 min.

### Thematic analysis

Table 1 summarises our results. Illustrative quotes are presented throughout. ‘ID-a’ denotes adolescents, ‘ID-p’ parents and ‘ID-h’ health professional.

**Table 1:** Results describing views on ACT and a potential trial presented as themes and subthemes

	Deductive themes	Inductive subtheme
Views on ACT	Acceptability	An extra possibility for those struggling
		Better than CBT-f
		Not suitable for everyone
		Accepting the word ‘acceptance’
	Feasibility	No more difficult to deliver but need specific training
		Timing of delivering ACT should be individualised
	Reasons why ACT could be efficacious	Pragmatism, acceptance and compassion are valued in chronic illness
		Cognitive defusion is less tiring but difficult to achieve
		Focussing on values helps to ‘get through’
		Addressing both psychological and physical needs
		Normalising difficulties is a beneficial life skill
Views on a trial	Barriers and facilitators to trial recruitment	Attitudes toward research
		Treatment fatigue
		Reluctance to be randomised

ACT, acceptance and commitment therapy; CBT-f, cognitive behavioural therapy-for-fatigue.

#### Acceptability

##### Extra possibility for those struggling

All 30 adolescents, parents and HCPs said ACT would ‘have value’ (ID-a). Adolescents saw it as an ‘extra possibility’ (ID-a) for managing CFS/ME, especially for those struggling. They felt therapy options were lacking, therefore an alternative treatment provided hope. HCPs welcomed ACT, agreeing ‘it’d be great to offer something else’ (ID-h).

What do we do with the kids who don’t recover? It’s a really big issue … (ID-h)

Ten of the 12 adolescents reported they would try ACT. Although, some were cautious because they were not ‘the biggest fan[s] of change’, they thought it was ‘worth trying’ (ID-a) if it provided a new possibility for treatment. Two participants said they would not try ACT because they did not need the treatment and would be ‘wasting a space for someone who needs it’ (ID-a) but recognised it could have been helpful for them earlier in their illness. See online supplemental table 2 for quotes.

##### Better than CBT-f

Two participants who had already received ACT thought it was more acceptable than CBT-f because it was ‘more gentle and kinder’ (ID-p), which was important for managing pain and fatigue. One adolescent found it ‘impossible’ (ID-a) to challenge thoughts in CBT-f because of the cognitive effort required, so preferred the ‘values’ and ‘person-centred’ focus in ACT.

CBT makes you feel like you’re constantly being challenged whereas ACT just feels like it’s more accepted […]whereas CBT is trying to push you back into your old [life] despite now having a chronically ill body. (ID-p and ID-a)

Others preferred ACT over CBT-f because it offered a ‘bigger picture’ and ‘journey approach’ (ID-p). One participant thought CBT-f was too focused on ‘nitty-gritty’ (ID-p) anxieties and could leave adolescents stuck in the past. They preferred how ACT, compared with CBT-f, has ‘goal setting’ and ‘practical elements […] focussed on values […] to move forwards in a positive direction about looking at what motivates people’ (ID-p).

##### Not suitable for everyone

Parents said ACT sounded ‘scary’ (ID-p) or ‘confrontational’ (ID-p) for younger or timid children to dismiss thoughts (cognitive defusion), rather than challenge them. In contrast, some adolescents felt this fear could be overcome: ‘Just the initial thought is quite scary but then after some time working on it would be okay’ (ID-a). The emotional engagement required for discussing values was felt ‘too challenging for some people [because] talking about stuff that’s really important could upset them’ (ID-a). Some questioned whether ACT was sufficiently CFS-focussed: ‘[ACT is for] anxiety and depression … I’d like to be explained why it would be helpful in CFS’ (ID-a).

##### Accepting the word ‘acceptance’

HCPs had concerns parents might think ACT means ‘you’ve just got to deal with it’ (ID-p) and misunderstand ACT to be about ‘where you’re at now’ (ID-h), whereas it is ‘more about where you’re going, it’s still about moving things forward just through a slightly different approach.’ (ID-h). In their experience, parents were always searching for treatments and may find it hard to accept therapy advocating acceptance so thought the word ‘acceptance’ needed clarification.

It’s being really clear about what we mean by acceptance … that acceptance [is] of thoughts and commitment to that bigger life in terms of your values … but I think sometimes when people hear that word ‘acceptance’ it can feel like just putting up with things. (ID-h)

#### Feasibility

##### No more difficult to deliver but need specific training

All HCPs felt it would be feasible to deliver ACT as it wasn’t ‘any more difficult’ (ID-h) than current psychological therapies and is currently being used, just ‘less formally and without a label’ (ID-h). However, a need for specific training was identified because ‘CBT is a part of core training but ACT isn’t’ (ID-h).

##### Timing of delivering ACT should be individualised

However, HCPs disagreed about *when* ACT should be offered or delivered. Some said at 12 months was not appropriate because patients may not have attended sufficient appointments by 12 months due to waiting times: ‘[treatment] is a year but our actual clinical contact with them is probably only six months’ (ID-h). They felt ACT would be more suitable for those ‘stuck’ (ID-h) after initial treatments, regardless of how long that took. Others felt ACT would be ‘beneficial from the get-go’ (ID-h) and should be offered from the beginning, not only at 12 months.

Adolescents’ opinions differed about whether ACT should be delivered after or alongside current treatments. For some, ‘doing the activity management and CBT [simultaneously] was too much’ (ID-p), especially while coming to terms with the diagnosis and ‘losing’ their former life. Other adolescents reflected how their mood was inevitably affected by CFS/ME and thought psychological treatment alongside AM/GET would be useful. Adolescents and parents repeatedly described the importance of preventing comorbid mood disorders in CFS/ME.

[CFS/ME] should be looked at more holistically and [ACT] offered not just if you’re struggling with your mental health but more as a starting point. (ID-p)

All participants agreed that the decision *if* and *when* to offer ACT should be a clinical decision ‘on an individual basis’ (ID-h) because ‘everyone’s different, […] what suits one person doesn’t suit another’ (ID-p).

#### Reasons why ACT could be efficacious

##### Pragmatism, acceptance and compassion are valued in chronic illness

Participants talked about ACT being pragmatic, realistic and accepting. They noted how thoughts and feelings around CFS/ME were valid and grounded in true events or understandable anxieties, so it was unhelpful to challenge thoughts by ‘changing being chronically ill to a happy thought’ (ID-a). Adolescents felt compassionate acceptance was a more appropriate approach for managing the loss and grief associated with CFS/ME, than ‘constantly telling them to challenge feelings and distract themselves from [thoughts]’ (ID-p).

##### Cognitive defusion is less tiring but difficult to achieve

Some adolescents expressed stepping away from thoughts (cognitive defusion) was a good tactic for dealing with negative cognitions and ‘get on with stuff’ (ID-a) because constantly filtering negative thoughts exacerbated fatigue. However, some thought dismissing thoughts was too difficult. They were unsure how to subsequently deal with dismissed thoughts: ‘I’d be all … what … like where … what am I supposed to do with [the thought] … just leave it?’ (ID-a).

##### Focusing on values helps to ‘get through’

Adolescents described losing ‘core values’ (ID-a) and thought ACT’s focus on values would be useful. They liked the practical element of committed action to values to help them ‘get through’ their illness (ID-a).

##### Addressing both psychological and physical needs

Families felt ACT recognised the wide-ranging health and social impacts of CFS/ME. Adolescents liked ACT’s holistic ‘universal’ (ID-a) approach to addressing both their ‘psychological condition, but also [ACT] helps you accept your physical one too’ (ID-a).

##### Normalising difficulties is a beneficial life skill

Parents thought that ‘normalising difficulties’ in ACT was helpful to understand worries and setbacks as part of ‘the human condition’ (ID-p) and felt that ‘we would all benefit from’ (ID-p) these life skills. HCPs agreed that normalising difficulties is especially important for managing CFS/ME in teenagers because they ‘struggle with feeling weird and unique’ (ID-h).

See online supplemental table 3 for illustrative quotes for theme 3.

#### Barriers and facilitators of trial recruitment

##### Attitude toward research

Seven of ten adolescents who said they would try ACT, said they would consent to an RCT. A key facilitator to recruitment was appreciating benefits of research. Participants expressed wanting to help others, even if the trial didn’t benefit them directly: ‘it’s not necessarily doing it for right now, it’s doing it for the longer-term’ (ID-p). Five participants had previously participated in trials, so had insight into research involvement.

##### Treatment fatigue

Two adolescents said they would not consent to an RCT because they felt de-motivated and new treatments were ‘passed [them] now’ (ID-a). HCPs also recognised that some might feel negative about another treatment because they ‘had tried everything’ (ID-h).

##### Reluctance to be randomised

Most understood randomisation was necessary for a trial. However, some were reluctant, stating that one RCT arm would suit them better, so if they got the opposite arm it might affect their engagement or belief in treatment efficacy. While most parents also agreed to randomisation, one would prefer if their child could ‘have the chance to do the other [arm] afterwards […] so if [they] can [receive] both [treatments], then that would be ideal’ (ID-p). Similarly, adolescents who found randomisation unacceptable said they might take part if they could subsequently receive the therapy they had not received in the trial.

See online supplemental table 4 for illustrative quotes for theme 4.

## Discussion

All participants said ACT was acceptable, and most adolescents would partake in an RCT. Parents and adolescents thought ACT was suitable for those with persistent CFS/ME symptoms because of its pragmatic and compassionate approach. Issues with delivering ACT and an RCT were discussed, including: extra training required for psychologists; timing of when ACT should be offered and concern that patients might confuse ‘acceptance’ with ‘giving up’.

Strengths of this study include: multi-perspective views from three participant groups; interviewing adolescents with a variety of ages and illness durations; good engagement (only one adolescent declined to participate); and recruiting from the pool of adolescents who would be eligible for an RCT. Limitations are that: participants provided opinions based on information about what ACT would involve rather than actually undergoing treatment; participants were likely biased toward being engaged in treatment and research which could overestimate acceptability of ACT and the proportion who would consent to a trial; few (four) males were interviewed; not all eligible patients in the service were contacted as sampling was opportunistic; and recruitment was from one UK paediatric specialist CFS/ME service, so results may not be generalisable to all eligible patients, males or other centres.

Our findings are consistent with results from a feasibility study with adolescents with functional somatic syndromes, where 90.5% completed group-based ACT and all would recommend it to a friend.^21^ In our study, some adolescents appeared to have a treatment preference for ACT or treatment-as-usual. This should be borne in mind when designing a trial.

Our study found that participants wanted pragmatic and values-focussed strategies in treatment, which is consistent with research on ACT in paediatric chronic pain,^9^ where the core elements of ACT (ie, ‘functional contextualism’^22^ to facilitate behaviour in line with personal values and goals^15^) have demonstrated efficacy.^23^ Adolescents highlighted the loss of their core-values during their illness, so perhaps values-based treatment serves as a motivational factor.^24^ They said a compassionate approach was also needed to address the grief and loss of sense-of-self which is common in CFS/ME.^25^ Similarly, they expressed the need for treatment that validates their thoughts, rather than challenges them. This is a key difference between how ACT and CBT-f approach cognitions^14 15^ and might be why some participants said they preferred ACT to CBT-f. While CBT-f also enhances acceptance,^26^ its centrality in ACT is unique.

Comparable to adult CFS/ME literature,^26–30^ our study identifies ‘acceptance’ as fundamental for being able to enjoy life while affected by CFS/ME. Although this is common to chronic illness,^31^ CFS/ME presents particular challenges related to stigma, contested diagnosis and uncertain aetiology.^32^ In adults, it has been suggested that acceptance should be targeted before commencing other treatment, to maximise clinical benefit,^27^ aligning with opinions of some participants in this study who proposed ACT should be offered at the beginning of treatment.

## Conclusion

This work suggests ACT is acceptable and most adolescents and parents would consent to randomisation for an RCT. Given patients and HCPs feel there is a lack of options for those who have not yet fully recovered after receiving currently evidenced treatments, we recommend further work to develop a pilot study of ACT to inform an effectiveness RCT. Issues raised for designing an RCT of ACT included: extra training required for psychologists; clear explanations to patients and parents that ‘acceptance’ is not synonymous with ‘giving up’; timing of when ACT should be offered; and consideration of trial design as some adolescents had a treatment preference for ACT versus treatment-as-usual.

## Data Availability

Data are available upon reasonable request. Qualitative data can be requested provided the request does not breach anonymity protocol.
