# Correction for ‘*qualitative study of the acceptability and feasibility of acceptance and commitment therapy for adolescents with chronic fatigue syndrome*’

**DOI:** 10.1136/bmjpo-2021-001139corr1

**Published:** 2024-06-03

Clery P, Starbuck J, Laffan A, *et al*. Qualitative study of the acceptability and feasibility of acceptance and commitment therapy for adolescents with chronic fatigue syndrome. *BMJ Paediatrics Open* 2021;5:e001139. doi:10.1136/ bmjpo-2021-001139

This article has been corrected since it was published online. A new funder information “Wellcome funder grant number 204813/Z/16/Z” has been added to the article.

